# Hematologic reference intervals of Danish sows at mid-gestation

**DOI:** 10.1186/s13028-019-0451-7

**Published:** 2019-03-27

**Authors:** Sheeva Bhattarai, Tore Framstad, Jens Peter Nielsen

**Affiliations:** 1 Department of Veterinary and Animal Sciences, Faculty of Health and Medical Sciences, University of Copenhagen, Grønnegårdsvej 2, 1870 Frederiksberg C, Denmark; 2 Department of Production Animal Clinical Sciences, Faculty of Veterinary Medicine, Norwegian University of Life Sciences, Campus Adamstuen, 0033 Oslo, Norway

**Keywords:** Anemia, Hemoglobin, Iron, Pig, Pregnancy

## Abstract

Hematologic reference intervals are useful tools for interpretation of laboratory results in swine practice and research. Until now, there were no hematologic reference intervals established for gestating Danish sows and those established in other countries are either out-dated, produced using very few sows or incomplete with few variables. In the past few decades there have been significant increases in litter size due to breeding, as well as the development of new analytical procedures for hematology and statistical procedures for calculating reference intervals. Therefore, hematologic reference intervals representing current pig production and technology are greatly needed. The main objective of this study was to provide updated and complete hematologic reference intervals for Danish sows at mid-gestation. Blood samples were collected at mid-gestation from 248 sows belonging to five commercial herds in Denmark and the samples were analysed for several hematologic variables using the Advia 2120i Hematology System. The reference intervals were calculated according to the guidelines of American Society for Veterinary Clinical Pathology. The reference interval for hemoglobin concentration was 103.1–145.0 g/L. For red blood cells, hematocrit and mean corpuscular volume, the 95% reference intervals were 4.98–7.50 × 10^12^/L, 0.32–0.47 L/L, 57.0–69.3 fL, respectively. The reference intervals established in this study are different from those previously reported in the literature. These reference intervals add to the current knowledge and will be useful in the prevention, diagnosis and treatment of several disease conditions in gestating sow populations.

## Findings

Hematologic variables are important tools for herd veterinarians and researchers for diagnosing disease or interpretation of laboratory results. Until now very few hematologic variables have been used in swine practice because of the limited availability of reference intervals.

There are very few studies [1–3] that report hematologic reference intervals of gestating sows. There are no studies reporting reticulocyte variables in sows and we have recently shown that hematologic variables other than hemoglobin, namely reticulocyte variables indicate early stage iron deficiency in piglets [4]. There have been tremendous changes in genetics and performance of sows in particular relation to litter size, analytical procedures for hematology and statistical procedures for calculating reference intervals. Therefore, there is a need for more recent reference intervals of complete hematologic variables including reticulocyte variables derived from current production and statistical procedures. Reference intervals produced for sows may vary within and between regions due to differences in breeding, feeding and other management procedures. Moreover, the reference intervals calculated for non-gestating sows may not be used for gestating sows [1], as several changes occur in the blood profile of sows during gestation. During mid-gestation, the hematological profile of sows might begin to change in order to supply the demand of rapidly growing fetuses. Therefore this study aimed at providing hematologic reference intervals for Danish sows at mid-gestation.

Five commercial sow herds with a minimum herd size of 1000 sows were selected for the study based on convenience in cooperation with a specialized veterinary pig practice. In all herds, the sows were group-housed and were free from clinical disease based on observation. None of the farms used additional iron supplementation other than the iron present in feed. The standard recommendation of iron for pregnant sows is 80 mg/kg [5]. From each herd, sows (Landrace × Yorkshire) at mid-gestation (56–70 days of gestation) were randomly selected. Fifty sows were sampled from each of the three herds while 47 and 51 sows were sampled from the other two herds, respectively. Blood was withdrawn into 10 mL EDTA tubes from the jugular vein of the snare-restrained sows and analysed within 24 h.

The blood samples were analyzed for hematologic variables; hemoglobin concentration (Hb), erythrocyte count (RBC), white blood cell count WBC (both peroxidase method and basophil method), neutrophils (absolute count and percentage), lymphocytes (absolute count and percentage), large unstained cells (LUC) (absolute count and percentage), monocytes (absolute count and percentage), eosinophils (absolute count and percentage), basophils (absolute count and percentage), red blood cell distribution width (RDW), hemoglobin distribution width (HDW), hematocrit (HCT), mean cell volume (MCV), mean corpuscular hemoglobin (MCH), mean cell hemoglobin concentration (MCHC), and corpuscular hemoglobin concentration mean (CHCM). Reticulocyte indices were also analyzed which included reticulocyte count (absolute and relative), reticulocyte hemoglobin content (Chr), reticulocyte corpuscular hemoglobin concentration mean (CHCMr), mean reticulocyte cellular volume (MCVr), reticulocyte red cell distribution width (RDWr), and reticulocyte hemoglobin distribution width (HDWr).

Hemoglobin, MCHC, CHCM, HDW, CHCMr and HDWr values were converted from mmol/L to g/L by multiplying by a factor 16.11 for ease of comparison with other studies. Hematology testing was done using the Advia 2120i Hematology System (Siemens Healthcare Diagnostics Inc. Tarrytown, NY 10591, USA) at the Veterinary Diagnostic Laboratory, Department of Veterinary Clinical Sciences, University of Copenhagen.

As Hb is the widely available hematologic variable for anemia diagnosis, sow with Hb value less than 100 g/L was removed from the analysis considering the sow as anemic [6] as done previously [7]. In total, nine sows were excluded due to this leaving a total of 239 sows. According to the American Society for Veterinary Clinical Pathology (ASVCP) guidelines, mean, median, first quartile (Q1), median (Q2) and third quartile (Q3) were calculated using PROC MEANS procedure in SAS 9.4 (SAS institute Inc, Cary, NC, USA). The difference between Q3 and Q1 was calculated as an interquartile range (IQR). Each of the variables was checked for the presence of extreme outliers and those outliers were removed from the analysis. Extreme outliers were defined as the values that were < Q1 − 3IQR or > Q3 + 3IQR [8]. Whenever any extreme outlier was detected, the sow was excluded from further analysis. Subsequently, 16 sows were excluded due to the presence of outliers in one or more of the hematologic variables: HDW (n = 3), absolute reticulocyte count (n = 5), reticulocyte percentage (n = 5), MCVr (n = 3), RDWr (n = 3), CHCMr (n = 3), HDWr (n = 3), Chr (n = 3), absolute neutrophil count (n = 2), absolute and percentage eosinophil count (n = 3) and absolute LUC count (n = 1). This resulted in a final data set consisting of 223 sows. The 95% percentile reference intervals (2.5th and 97.5th percentile) method was used to determine the reference intervals because of sample size > 120 as recommended by ASVCP [9]. The reference intervals with 90% confidence limits were calculated with Reference Value Advisor (V.2.1) freeware, a set of macroinstructions for Microsoft Excel [10].

The reference intervals of erythrocyte and leucocyte variables including mean, median and range are presented in Tables 1 and 2, respectively.

**Table 1:** Erythrocyte variables’ reference intervals of Danish sows at mid-gestation

Variable	Unit	N	Mean	Median	Range (min–max)	Reference limits with 90% confidence limits (CL)	
						Lower (90% CL)	Upper (90% CL)
Hemoglobin	Mmol/L	223	7.5	7.4	6.3–10.5	6.4 (6.3–6.4)	9.0 (8.6–9.8)
	g/L	223	120.3	119.2	101.5–169.1	103.1 (101.5–103.1)	145.0 (138.5–157.8)
RBC	10^12^/L	223	6.14	6.07	4.86–8.99	4.98 (4.88–5.12)	7.50 (7.21–8.71)
Hematocrit	L/L	223	0.39	0.38	0.32–0.55	0.32 (0.32–0.33)	0.47 (0.45–0.52)
MCV	fL	223	63.4	63.5	55.6–70.2	57.0 (56.3–57.9)	69.3 (68.3–70.0)
MCH	fmol	223	1.22	1.23	1.08–1.36	1.09 (1.09–1.10)	1.34 (1.34–1.36)
MCHC	Mmol/L	223	19.27	19.28	18.21–21.02	18.37 (18.24–18.54)	20.31 (19.98–20.64)
	g/L	223	310.43	310.60	293.36–338.63	295.94 (293.84–298.67)	327.19 (321.87–332.51)
CHCM	Mmol/L	223	19.48	19.51	18.19–20.61	18.64 (18.42–18.76)	20.35 (20.10–20.55)
	g/L	223	313.82	314.30	293.04–332.02	300.29 (296.74–302.22)	327.83 (323.81–331.06)
RDW	%	223	15.7	15.7	13.7–18.5	14.4 (14.2–14.5)	17.2 (17.1–18.4)
HDW	Mmol/L	223	1.00	0.99	0.86–1.28	0.89 (0.86–0.91)	1.15 (1.13–1.25)
	g/L	223	16.11	15.94	13.85–20.62	14.33 (13.85–14.66)	18.52 (18.20–20.13)
CHr	fmol	223	1.32	1.32	1.13–1.51	1.19 (1.17–1.21)	1.46 (1.44–1.49)
Retic %	%	223	0.5	0.4	0.1–1.4	0.2 (0.1–0.2)	1.0 (1.0–1.3)
Retic	10^9^/L	223	29.8	25.8	6.4–93.0	9.8 (7.6–11.3)	73.6 (63.4–82.6)
MCVr	fL	223	78.7	78.7	67.1–91.2	69.2 (67.5–70.8)	88.1 (86.1–89.8)
RDWr	%	223	20.1	19.8	13.8–31.1	14.7 (13.9–14.9)	29.0 (27.2–30.5)
CHCMr	Mmol/L	223	17.04	17.01	15.85–18.72	16.13 (15.91–16.19)	18.29 (18.06–18.66)
	g/L	223	274.51	274.03	255.34–301.57	259.58 (256.31–260.82)	294.65 (290.94–300.61)
HDWr	Mmol/L	223	2.33	2.29	1.38–3.91	1.58 (1.49–1.67)	3.32 (3.10–3.43)
	g/L		37.53	36.89	22.23–62.99	25.77 (24.00–26.90)	53.48 (49.94–55.25)

The number of digits after decimal point is presented as received from analyzer to ensure that precision level of the test is correctly presented. HCT values were obtained in three digits after decimal points which are rounded to two digits

*RBC* red blood cell count, *MCV* mean corpuscular volume, *MCHC* mean cell hemoglobin concentration calculated based on Hb, MCV and RBC, *CHCM* mean cell hemoglobin concentration directly measured as cell-by-cell analysis, *MCH* mean corpuscular hemoglobin, *MPV* mean platelet volume, *RDW* red blood cell distribution width, *HDW* hemoglobin distribution width, *MCVr* reticulocyte cellular volume, *CHCMr* mean reticulocyte corpuscular hemoglobin concentration, *Chr* reticulocyte hemoglobin content, *RDWr* reticulocyte red cell distribution width, *HDWr* reticulocyte hemoglobin distribution width, *Min* minimum, *Max* maximum

**Table 2:** Leukocyte variables’ reference intervals of Danish sows at mid-gestation

Variable	Unit	N	Mean	Median	Range (min–max)	Reference limits with 90% confidence limits (CL)	
						Lower (90% CL)	Upper (90% CL)
WBCP	10^9^/L	223	15.79	15.57	8.67–23.64	10.12 (9.67–10.82)	22.24 (21.49–22.88)
WBCB	10^9^/L	223	15.09	14.09	8.01–22.89	9.81 (9.12–10.81)	21.35 (20.73–22.46)
Neutrophils	10^9^/L	223	5.97	5.58	2.06–11.95	3.04 (2.23–3.34)	10.94 (9.73–11.64)
Neutrophils	%	223	38.4	38.0	22.6–57.8	26.4 (24.4–27.9)	55.1 (52.8–57.5)
Eosinophils	10^9^/L	223	1.16	1.05	0.07–3.75	0.19 (0.11–0.38)	2.81 (2.50–3.18)
Eosinophils	%	223	7.4	6.9	0.5–19.0	1.5 (0.9–2.9)	16.1 (13.6–17.7)
Basophils	10^9^/L	223	0.13	0.12	0.04–0.32	0.05 (0.05–0.06)	0.24 (0.22–0.28)
Basophils	%	223	0.8	0.8	0.4–1.7	0.5 (0.4–0.5)	1.4 (1.2–1.6)
Monocytes	10^9^/L	223	0.64	0.60	0.14–1.26	0.32 (0.24–0.38)	1.15 (1.02–1.25)
Monocytes	%	223	4.2	4.0	1.4–7.4	2.7 (2.2–2.8)	6.4 (6.1–7.3)
Lymphocytes	10^9^/L	223	7.28	7.18	3.50–12.52	4.56 (3.73–4.86)	11.25 (9.91–11.98)
Lymphocytes	%	223	47.8	48.2	30.4–68.2	33.9 (31.9–34.6)	59.9 (58.6–64.0)
LUC	10^9^/L	223	0.22	0.22	0.02–0.68	0.04 (0.02–0.05)	0.48 (0.44–0.61)
LUC	%	223	1.4	1.4	0.1–3.5	0.3 (0.2–0.4)	2.8 (2.6–3.5)

The number of digits after decimal point is presented as received from the analyser

*WBCP* white blood cell determined by peroxidase method, *WBCB* white blood cell determined by basophile method, *LUC* large unstained cells, *Min* minimum, *Max* maximum

The present study reported hematologic reference intervals for sows at mid-gestation. There are many factors that affect reference intervals. These factors include selection criteria of herds and animals, breed, age, stage of reproductive cycle, management conditions, season [1], blood-sampling strategies, duration between sampling and analysis, method of blood analysis, and method of data analysis. In this study, data from five herds were combined for the calculation of reference intervals in order to account for herd effect variations in Denmark. None of the farms used iron supplementation for the sows which could otherwise affect some blood variables. All the samplings were done during the winter to avoid seasonal variation if any and at the same gestational stage.

A review [11] has highlighted the importance of distinguishing physiological regulation by the body during pregnancy and pathological deviations from the normal while interpreting blood variables. The discussion on time-dependent reference intervals within veterinary medicine is on-going and our study contributes to the issue. Pregnancy in sows is a special physiological condition in which adjustment in hemopoiesis takes place for the growth and development of the fetus. Several changes in blood variables occur as the pregnancy progresses. In humans, the blood volume increases during pregnancy with decreases in Hb concentration [12] and hematocrit, increased reticulocyte numbers and increased MCV all depending on iron availability [13]. A similar change may occur in sows which explains the need of reference intervals for gestating sows.

The reported lower reference limits of this study are different from the reference values reported in Canada [3]. In our study sows were excluded according to defined criteria for anemia. The upper reference value of Hb is extremely high (170 g/L) in the Canadian study compared to the current study. Similarly, upper reference limits of RBC, MCHC, WBC, neutrophil %, lymphocyte %, lymphocyte absolute, monocyte %, monocyte absolute, basophil % and basophil absolute are higher in that study [3]. Upper reference limits of MCV, absolute neutrophil count, absolute eosinophil count and eosinophil % are a bit high in our study while HCT values are similar in both studies. From 2013, all sows in Denmark are kept in groups by legal requirement starting from 4 weeks after service to 1 week before the expected date of farrowing. This might be a risk factor for parasitic infestation resulting in higher eosinophil numbers than found in other studies [1, 3]; this needs further investigation. However, a previous study [14] found a very low prevalence of parasitic infection in Denmark.

Better hematologic status in the sows reported by Friendship [3] is not surprising because the productivity of sows has dramatically changed over the last decades. The litter size in Denmark has increased continuously throughout the last 20 years from 12 to more than 18 piglets per litter [15]. The amount of iron required for fetal growth is therefore much higher now than in the past which requires sufficient iron stores in sows.

We recognize some limitations which should be considered while interpreting the reference intervals of this study. The study was done in apparently healthy sows, but sub-clinical infections and diseases were not considered. Furthermore, platelet variables could not be reported because of platelet aggregation as reflected by pseudothrombocytopenia. However, microscopic examination for detection of platelet aggregation was not done. It has been reported in human studies that platelet aggregation occurs with the use of anticoagulant EDTA [16, 17], which was used in the current study.

In conclusion, this study generated up-to-date hematologic reference intervals for gestating sows that represent pig production with highly prolific sows. These reference intervals will be useful for herd veterinarians and researchers to diagnose a disease condition or better understand changes that occur in sows during the gestational period.

## Abbreviations

ASVCP: American Society for Veterinary Clinical Pathology
CHCM: corpuscular hemoglobin concentration mean
CHCMr: reticulocyte corpuscular hemoglobin concentration mean
Chr: reticulocyte hemoglobin content
CL: confidence limits
EDTA: ethylenediamine tetraacetic acid
Hb: hemoglobin
HCT: hematocrit
HDW: hemoglobin distribution width
HDWr: reticulocyte hemoglobin distribution width
IQR: interquartile range
LUC: large unstained cells
MCH: mean corpuscular hemoglobin
MCHC: mean cell hemoglobin concentration
MCV: mean cell volume
MCVr: mean reticulocyte cellular colume
Q1: first quartile
Q2: median (second quartile)
Q3: third quartile
RBC: red blood cells (erythrocyte count)
RDW: red blood cell distribution width
RDWr: reticulocyte red cell distribution width
WBC: white blood cell
